# Indigenous oil-degrading bacteria more efficient in soil bioremediation than microbial consortium and active even in super oil-saturated soils

**DOI:** 10.3389/fmicb.2022.950051

**Published:** 2022-08-01

**Authors:** Nedaa Ali, Majida Khanafer, Husain Al-Awadhi

**Affiliations:** Microbiology Program, Department of Biological Sciences, Faculty of Science, Kuwait University, Kuwait City, Kuwait

**Keywords:** bioremediation, bioaugmentation, heavy oil-pollution, self-cleaning, hydrocarbonoclastic bacteria

## Abstract

A microbial consortium of the hydrocarbonoclastic bacterial species, comprising *Actinotalea ferrariae, Arthrobacter ginsengisoli, Dietzia cinnamea, Dietzia papillomatosis*, and *Pseudomonas songnenensis*, isolated from oil-saturated desert soil did not consume more oil in batch cultures than the individual species with the maximum oil consumption. In oil-polluted desert soil microcosms, the rate of oil removal in the soil samples bioaugmented with the microbial consortium was similar to the rate of oil removal in the unbioaugmented ones through a 6-month bioremediation experiment. Although the composition of hydrocarbonoclastic bacterial communities in the unbioaugmented and bioaugmented soil samples was different, the predominant bacterial species during most of the months were the same. Toward the end of the bioremediation experiment, *Ar. ginsengisoli* prevailed in both soil samples, suggesting its important role in oil removal. Self-cleaning proceeded in desert soil samples artificially polluted with 1, 10, 20, and 30% of crude oil and incubated at 30 °C for 6 months. Oil was removed effectively at rates reaching 73.6 and 69.3% in the soils polluted with 1 and 10% oil concentrations, respectively, and reached 50% in desert soils polluted with 20 and 30% oil concentrations. The bacterial numbers increased in all soil samples from hundreds of thousands per gram of soil samples at time zero to millions and tens of millions per gram of soil samples after 6 months. It was concluded that bioaugmenting oil-polluted soil samples with microbial consortium of hydrocarbonoclastic bacterial species with high oil removal potential did not drastically enhance oil bioremediation and that even in super oil-saturated soils, indigenous oil-degrading bacteria will prevail and effectively contribute to oil removal from the surrounding environment.

## Introduction

Kuwait is a major oil supplier and holds approximately 7% of global oil reserves. Kuwait experienced a number of oil spills during tanker loading at oil terminals where oil comprises around 95% of the exports. A major oil spill occurred in 1991 (Second Gulf War) when the Iraqi forces, during their withdrawal, blasted more than 800 wells of which more than 600 wells were burned. Oil from about 50 wells gushed onto land and covered about 200 km^2^ of the ground forming around 250 oil lakes with a total oil volume estimated to be 10–20 million tons (Salam, 1996; Omar et al., 2000; Linden et al., 2004).

Crude oil consists of a mixture of hydrocarbons (aliphatic, cycloaliphatic, and aromatic) and non-hydrocarbons (resins and asphaltenes) components, most of which have low water solubility. Crude oil penetrates the soil and its constituents bind to soil components changing its chemical and physical properties and altering the living environment therein (Sui et al., 2021; Wang et al., 2021). Some of the oil hydrocarbons, particularly the aromatic compounds, are toxic (Wang et al., 2021) and difficult to be degraded, so persist in the soil (Andreoni and Gianfreda, 2007) and become hazardous to living beings.

Oil removal by bioremediation approaches is preferred over the physical (e.g., (incineration) and chemical approaches (e.g., chemical oxidation), because it is more economic and eco-friendly (Rosenberg, 1993; Kuiper et al., 2004). Although the physical and chemical methods are effective in oil removal, they have hazardous consequences. For example, incineration causes air pollution and reduces the carbon and organic content of the soil, and chemical oxidation inhibits microbial growth in the soil (Sui et al., 2021). Through bioremediation technology, microorganisms owing to their ability to utilize oil as their sole source of carbon and energy are used to degrade oil into harmless products (CO_2_ and water). This technology involves two strategies: bioaugmentation, which implies the introduction of efficient oil-degrading microorganisms into polluted sites (Kuiper et al., 2004; Szulc et al., 2014), and biostimulation, in which the activities of indigenous oil-degrading microorganisms are enhanced through the addition of nutrients like nitrogen and phosphorous (Atlas and Bartha, 1998; Radwan, 2009). Self-cleaning implies the ability of soil indigenous microorganisms to remove oil spontaneously.

It is noteworthy to point out that most of the bioremediation studies used low concentrations of crude oil reaching 7% (Hesnawi and Mogadami, 2013; Qiao et al., 2013; Goudarztalejerdi et al., 2015; Siles and Margesin, 2018; Castro Rodríguez et al., 2022). Vasilyeva et al. (2020) used oil concentrations of 5–15% and reported that bioremediation can be applied on oil concentrations less than 5% only due to toxicity. However, Benyahia et al. (2005), working with a biopile system, studied bioremediation of soils saturated with crude oil and reported a faster reduction in the initial oil concentration when the polluted soil was amended with commercial oil-degrading bacteria.

In our earlier study (Ali et al., 2020a,b), we showed that self-cleaning proceeds in oil-saturated soils (17.3%, w/w oil), where indigenous bacteria capable of dealing with such high oil concentrations become enriched and involved in oil biodegradation. The objective of this study is to determine whether bioaugmenting oil-polluted soil with efficient oil-degrading bacteria isolated from oil-saturated soils would enhance the oil bioremediation process when introduced into oil-polluted soils. We are also aiming to determine whether self-cleaning would proceed in soils super-saturated with oil, i.e., oil concentrations > 17.3%.

## Materials and methods

### Construction of the consortium

Five hydrocarbonoclastic bacterial strains with a high potential to degrade crude oil isolated from oil-saturated (17.3%, w/w) Kuwaiti desert soil (Ali et al., 2020a,b) were selected to perform this study. The bacterial strains belong to the following genera: *Actinotalea ferrariae, Arthrobacter ginsengisoli, Dietzia papillomatosis, Dietzia cinnamea*, and *Pseudomonas songnenensis*. A common inoculum for each individual strain was prepared by suspending a loopful of fresh biomass in 5 mL of mineral medium (Sorkhoh et al., 1990), and the OD_600_ was adjusted to 1.0. The consortium consisted of equal proportions of each suspension with the total volume adjusted to 5 mL with the mineral medium. The mineral medium (Sorkhoh et al., 1990) included the following chemicals (g^−1^): 5.0 NaNO_3_, 0.56 KH_2_PO_4_, 0.86 Na_2_HPO_4_, 0.17 K_2_SO_4_, 0.37 MgSO_4_.7H_2_O, 0.007 CaCl_2_.H_2_O, and 25 mL L^−1^ of a trace element solution consisting of (gL^−1^) 2.32 CuSO_4_.5H_2_O, 0.39 Na_2_MoO_4_. 2H_2_O, 0.66 KI, 1.0 EDTA, 0.4 FeSO_4_.7H_2_O, and 0.004 NiCl_2_.6H_2_O. The pH was adjusted to 7.

### Oil consumption by the consortium in batch culture

To study the potential of the bacterial consortium for oil removal in batch culture, 100-mL screw-capped flasks containing 25 mL aliquots of mineral medium (Sorkhoh et al., 1990) amended with 5 or 10% (w/v) crude oil, as a sole source of carbon and energy, were inoculated with 0.5-mL portions of the consortium. For comparison, parallel sets of flasks for each individual bacterial strain were inoculated with 0.5-mL portions of each individual bacterial suspension. Control aliquots contained uninoculated mineral medium. Triplicate flasks were prepared throughout. The cultures were incubated on an electrical shaker at 180 rpm and 30 °C for 10 d.

The residual oil in each aliquot was recovered by three successive extractions with 10-mL portions of pentane. The three extracts were combined, and the total volume was completed to 50 mL using pentane, and 1 μl of the sample was analyzed by gas-liquid chromatography (GLC) using an Agilent 7890A GLC (USA) system equipped with FID, a DB-5 capillary column (Agilent Technologies, USA) with He as a carrier gas. The initial oven temperature was set at 50 °C for 3 min, and then increased at 3 °C/min to 80 °C, at 8 °C/min to 256 °C, at 30 °C/min to 330 °C, and finally, this temperature was maintained for 11 min. Oil consumption was calculated in terms of the reduction values of total peak areas based on the total peak areas of the oil recovered from the control.

The bacteria in the consortium-inoculated flasks were counted following the dilution plate method using the mineral medium (Sorkhoh et al., 1990) solidified with agar (20 g L^−1^). Oil vapor, a sole source of carbon and energy, was supplied from a sterile filter paper saturated with oil fixed to the plate lid. For each dilution, three replicate plates were inoculated; the plates were sealed and incubated at 30 °C for 10 d.

### Soil bioremediation *via* bioaugmentation with the consortium

To study the potential of the microbial consortium for cleaning up desert soil samples polluted with crude oil, 3 kg of pristine desert soil collected from Mishrif, Kuwait, was artificially polluted with 45 g of Kuwaiti light crude oil (1.5 %, w/w). The polluted soil was divided equally into two glass trays: one was bioaugmented with 100 mL of the microbial consortium and the other was mixed with 100 mL of sterile water (unbioaugmented, control). The trays were kept exposed to open environmental conditions for 6 months. The soil samples were irrigated with 10% sterile water and mixed thoroughly 2–3 times a week. Samples were collected at time zero and monthly, up to 6 months, for monitoring the microbial communities and the residual crude oil.

The hydrocarbonoclastic microorganisms were counted following the dilution plate method using mineral medium (Sorkhoh et al., 1990) and oil vapor as a sole source of carbon and energy, as described above. The total number of colony-forming units (CFU) was counted, and representatives of the most predominant identical colonies (colors, shapes, sizes, margins, and consistencies) were isolated and purified. Pure isolates were identified by comparing their 16S-rRNA gene sequences with those of type strains in the GenBank database. For this purpose, the total genomic DNA of each isolate was extracted using PrepMan Ultra Sample Preparation Reagent (Applied Biosystems, USA), and the 16S-rRNA gene therein was amplified by using the universal primer pairs, GM5F (5'-CCT ACG GGA GGC AGC AG-3') and 907R (5'-CCG TCA ATT CMT TTG AGT TT-3') (Santegoeds et al., 1998), by polymerase chain reaction (PCR). A Veriti Thermal Cycler (Applied Biosystems, USA) following a touch-down protocol of initial denaturation at 95 °C for 5 min, annealing temperature starting at 65 °C and decreasing by 1 °C every cycle to 55 °C, at which additional 12 cycles were carried out, denaturation was carried out at 94 °C for 1 min, and primer extension was performed at 72 °C for 1 min. The final extension was done at 72 °C for 7 min. The PCR mixture consisted of the following components in a final volume of 25 μl of molecular water (Sigma): a puReTaq Ready-To-Go PCR bead (Amersham Biosciences, UK), 1 μl (25 ng) of DNA template, and 1 μl each of the universal primers (Ali et al., 2020a). The PCR products were purified using the QIA quick PCR purification kit (Qiagen, USA) following the manufacturer's instructions. The 16S-rRNA genes were partially sequenced by the BigDye version 3.1 Terminator Kit (Applied Biosystems, USA). The reaction mixture consisted of the following compounds in a final volume of 10 μl of molecular water: 20 ng of the DNA template, 2 μl of the BigDye Terminator ready reaction mix, 2 μl of the 5X sequencing buffer, and l μl primer of either 907R or GM5F. Labeling was completed in the Veriti Thermal Cycler (Applied Biosystems, USA) with one cycle performed at 96 °C for l min, and then 25 cycles of l min at 96 °C, 5 s at 50 °C, and 4 min at 60 °C. The DNA samples were processed in a 3130xl genetic analyzer (Applied Biosystems, USA) with sequencing analysis version 5.2 software (Applied Biosystems, USA). Sequences were subjected to basic local alignment search tool analysis with the National Center for Biotechnology Information (NCBI; Bethesda, MD, USA) GenBank database (Altschul et al., 1997).

For the determination of the residual oil, five random 1-g aliquots were harvested from the unbioaugmented and bioaugmented soil samples. Each aliquot was extracted three times with 5-mL portions of pentane. The three extracts were combined, and the total volume was completed to 20 mL with pentane. The residual oil in the combined extracts was determined using GLC following the same protocol as previously described. The amount of oil consumed was calculated in terms of the reduction values of total peak areas based on the total peak areas of the oil recovered from time zero samples.

### Bioremediation of super oil-saturated soils

In a parallel experiment, 100-g aliquots of pristine Kuwaiti desert soil samples in 1 L beakers were polluted artificially with 1, 10, 20, or 30% (w/w) crude oil. Control aliquots were kept oil-free. The beakers were sealed with double layers of Para film and were incubated at 30 °C. All samples received equal amounts of sterile water (10%) and were mixed thoroughly twice a week for aeration. Two samples were analyzed, one at time zero and the other after 6 months for the determination of the hydrocarbonoclastic communities and the residual oil following the same methods described above.

### Oil tolerance by pure isolates

Representative six pure cultures of the most predominant bacterial isolates grown in the presence of various oil concentrations (1–30%, w/w) for 6 months were tested for their oil tolerance and consumption capacities. A common inoculum (loopful of a fresh bacterial culture homogenized in 5 mL of sterile water) for each isolate was prepared. Aliquots of 0.5 mL of the inocula were inoculated in screw-capped flasks containing 25 mL of mineral media (Sorkhoh et al., 1990) and oil at different concentrations [1, 5, 10, 20, or 30% (w/v)]. The cultures were incubated on electrical shakers at 180 rpm and 30 °C for 10 d, after which bacterial numbers and oil consumption were determined. The bacteria were counted following the dilution plate method on the mineral medium (Sorkhoh et al., 1990) with crude oil as the sole source of carbon and energy. The residual oil was recovered and determined by GLC, where the oil consumption was calculated in terms of the reduction values of total peak areas based on the total peak areas of the oil recovered from uninoculated aliquots (control). The methods followed for bacterial counting and residual oil determination have been detailed above.

### Statistical analysis

The analyses were performed by Microsoft Excel 2007 (Microsoft, USA). Three replicates were done for each analysis, and the mean ± standard deviation values were calculated. The effect of bioaugementation on oil removal efficiency from polluted soil was statistically analyzed by independent *t*-test with the null hypothesis that bioaugmentation has no effect on oil removal from the polluted soil. A *p*-value of < 0.05 was the significance level for declaring statistical significance.

## Results

### Oil consumption by the consortium in batch culture

**Figure 1:**
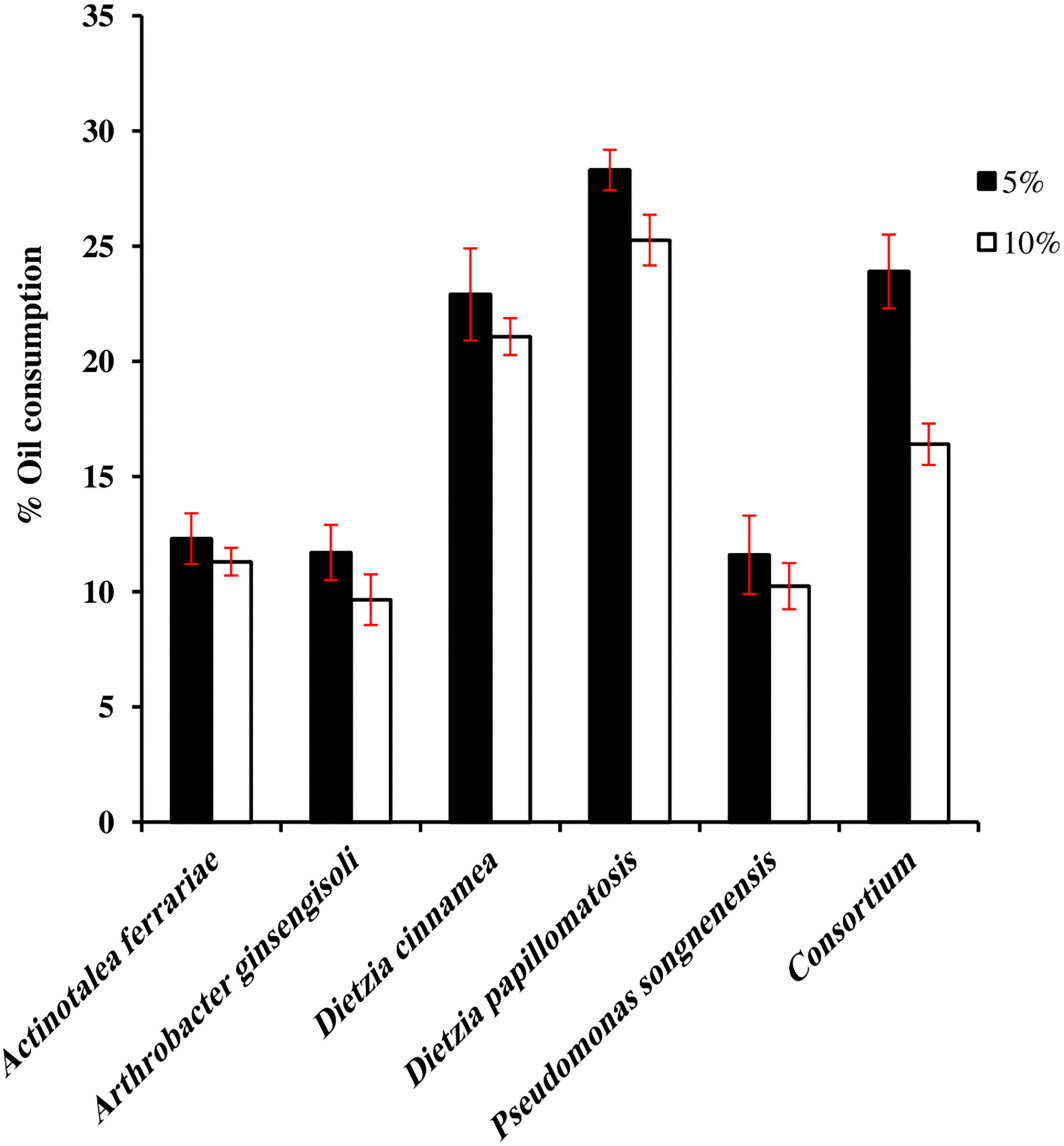
Oil consumption by individual hydrocarbonoclastic bacteria and consortium.

Figure 1 presents the oil-consumption potential of the individual bacterial strain and the consortium. *D. papillomatosis* showed the highest oil-consumption potential among all the other bacterial strains and the consortium as well. *D. papillomatosis* removed 28.3 and 25.3% of the oil when the initial concentration of oil in the medium was 5 and 10%; respectively, whereas the consortium removed 23.9 and 16.4% of the oil when the initial concentration of oil in the medium was 5 and 10%, respectively. Of the five bacterial strains constituting the consortium, *P. songnenensis* took over the absolute predominance with 12.8 × 10^7^ CFU mL^−1^ in the 5% oil-containing mineral medium; the CFU numbers of the other four strains were negligible at high dilutions in the dilution plate count method. In the 10% oil-containing mineral medium, *Ar. ginsengisoli* and *D. papillomatosis* shared the dominance with 16.6 × 10^7^ and 15.6 × 10^7^ CFU mL^−1^, respectively, whereas the counts of *P. songnenensis* decreased to 4.2 × 10^7^ CFU mL^−1^. No significant counts were recorded for *A. ferrariae* and *D. cinnamea*.

### Soil bioremediation *via* bioaugmentation with consortium

#### Number of hydrocarbonoclastic bacteria

The results in Table 1 show the number of hydrocarbonoclastic bacteria recorded during the 6-month study in both unbioaugmented and bioaugmented oil-polluted desert soils. The highest count of the colony-forming units (CFU, 739 × 10^5^ CFU g^−1^) in the unbioaugmented soil samples was recorded at the end of the first month of the bioremediation experiment. The CFU numbers decreased with time, reaching 39.7 × 10^5^ CFU g^−1^ at the end of the bioremediation process (6 months). An increase in the CFU numbers was recorded at the end of the fourth month (May), reaching 418.5 × 10^5^ CFU g^−1^. The highest CFU count in the bioaugmented soil samples (915 × 10^5^ CFU g^−1^) was recorded at time zero, after the immediate inoculation of the consortium; the CFU numbers then decreased with time reaching 42.9 × 10^5^ CFU g^−1^ at the end of the bioremediation experiment. The CFU numbers in the bioaugmented soil samples also showed an increase at the end of the fourth month (May), reaching 194.0 10^5^ CFU g^−1^. The independent *t*-test analysis showed no significant difference in the CFU numbers among the unbioaugmented and bioaugmented oil-polluted soil samples (*n* = 3, *p* > 0.05).

**Table 1:** Numbers of CFU of hydrocarbonoclastic bacteria in unbioaugmented and bioaugmented oil-polluted desert soil.

	**End of the month**	**Numbers of CFU**	
	**(min-max** °**C)**	**g**^−1^ **(x 10**^5^**)**	
		**Unbioaugmented**	**Bioaugmented**
		**soil**	**soil**
0	January (14–26)	420.0 ± 6.2	915.0 ± 12.7
1	February (19–24)	739.0 ± 14.2	577.0 ± 10.8
2	March (19–29)	98.5 ± 2.0	54.5 ± 0.2
3	April (26–37)	194.5 ± 1.3	154.5 ± 8.1
4	May (36–48)	418.5 ± 3.7	194.0 ± 3.3
5	June (43–51)	128.0 ± 1.6	23.5 ± 1.2
6	July (44–50)	39.7 ± 4.3	42.9 ± 1.9

± standard deviation values.

#### Dynamics of hydrocarbonoclastic bacterial communities

**Figure 2:**
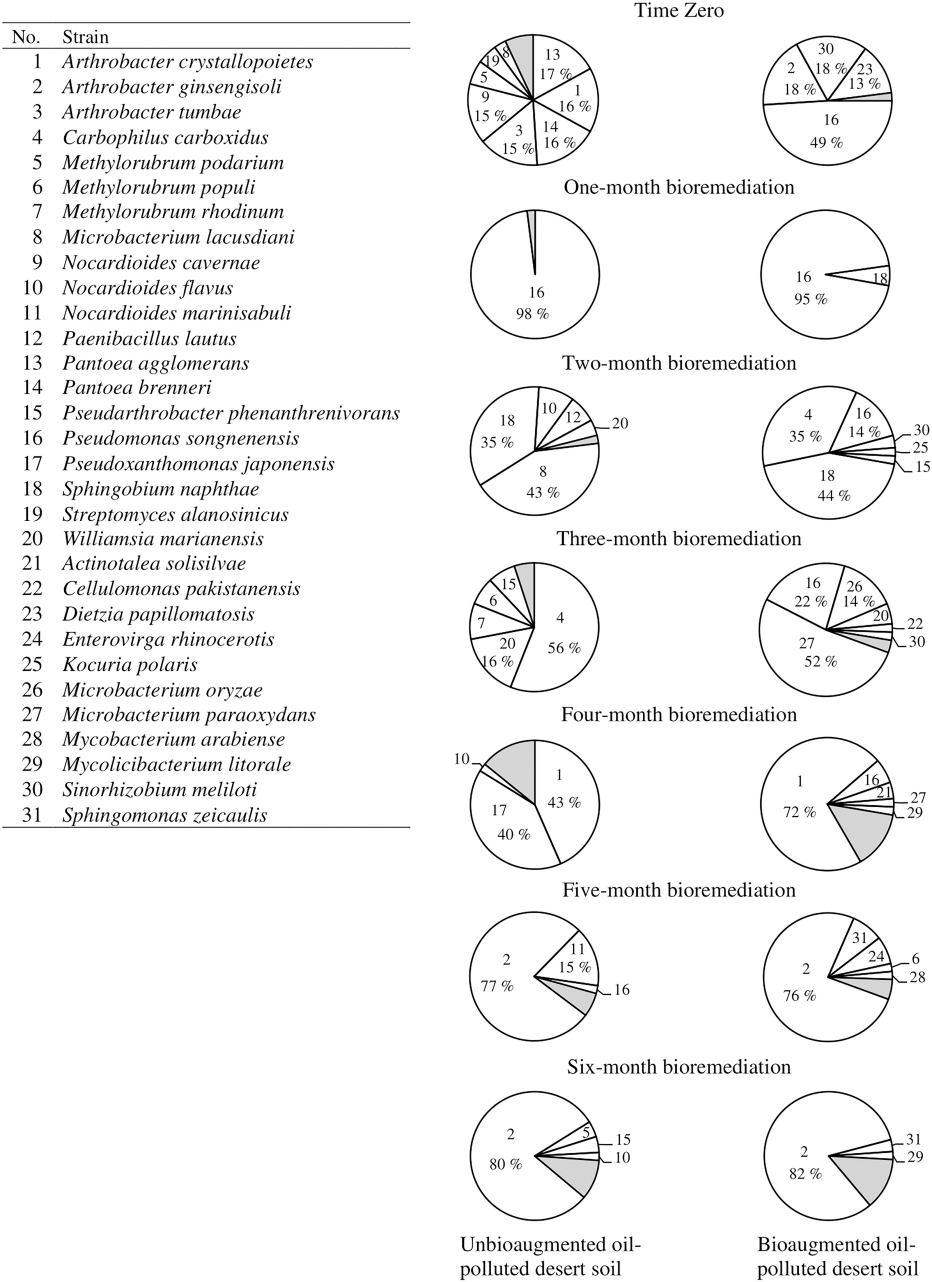
Dynamics of hydrocarbonoclastic microbial communities in the unbioaugmented and bioaugmented oil-polluted desert soil. Shaded areas comprise minor organisms (1–2% occurrence), refer to Supplementary Table S3 for their identification.

Figure 2 presents the composition of the hydrocarbonoclastic bacterial communities in the unbioaugmented and bioaugmented oil-polluted desert soils and the dynamics of these communities through the 6-month bioremediation experiment. At time zero, the predominant bacterial species in the unbioagumented soil were *Pantoea agglomerans* (17%), *Pantoea brenneri* (16%), *Arthrobacter crystallopoietes* (16%), *Arthrobacter tumbae* (15%), and *Nocardioides cavernae* (15%). At the end of the 1st month of the bioremediation experiment, *P. songnenensis* prevailed (98%). At the end of the 2nd month, *Microbacterium lacusdiani* and *Sphingobium naphthae* shared the predominance (43 and 35%, respectively). At the end of the 3rd month, *Carbophilus carboxidus* (56%) prevailed, and the number of *Williamsia marianensis* (16%) increased. At the end of the 4th month, *Ar. crystallopoietes* (43%) and *Pseudoxanthomonas japonensis* (40%) shared the predominance. At the end of the 5th month, *Ar. ginsengisoli* (77%) was predominant, followed by *Nocardioides marinisabuli* (15%). At the end of the 6th month (end of the bioremediation experiment), *Ar. ginsengisoli* (80%) predominated.

The bacterial community of the soil bioaugmented with microbial consortium showed the following pattern; at time zero, *P. songnenensis* (49%) was the predominant strain followed by *Ar. ginsengisoli* and *Sinorhizobium meliloti* (18 %, each) and *D. papillomatosis* (13%). At the end of the 1st month, *P. songnenensis* (95 %) prevailed and *S. naphthae* (5%) appeared. At the end of the 2nd month, *S. naphthae* (44%) and *C. carboxidus* (35%) shared the predominance over *P. songnenensis* (14%). At the end of the 3rd month, *Microbacterium paraoxydans* (52%) prevailed over *P. songnenensis* (22%) and *Microbacterium oryzae* (14%). At the end of the 4th month, *Ar. crystallopoietes* (72%) prevailed. At the end of the 5th and 6th months, *Ar. ginsengisoli* (76 and 82%, respectively) prevailed. Supplementary Table S1 presents the identities of the isolated hydrocarbonoclastic bacteria determined by comparing their 16S-rRNA genes with those of type strains in the GenBank database. The identified taxa had 99–100% similarity to their GenBank counterparts. The sequences were deposited in the GenBank under the accession numbers MN905376 - MN905452.

#### Oil removal by hydrocarbonoclastic bacterial communities

**Figure 3:**
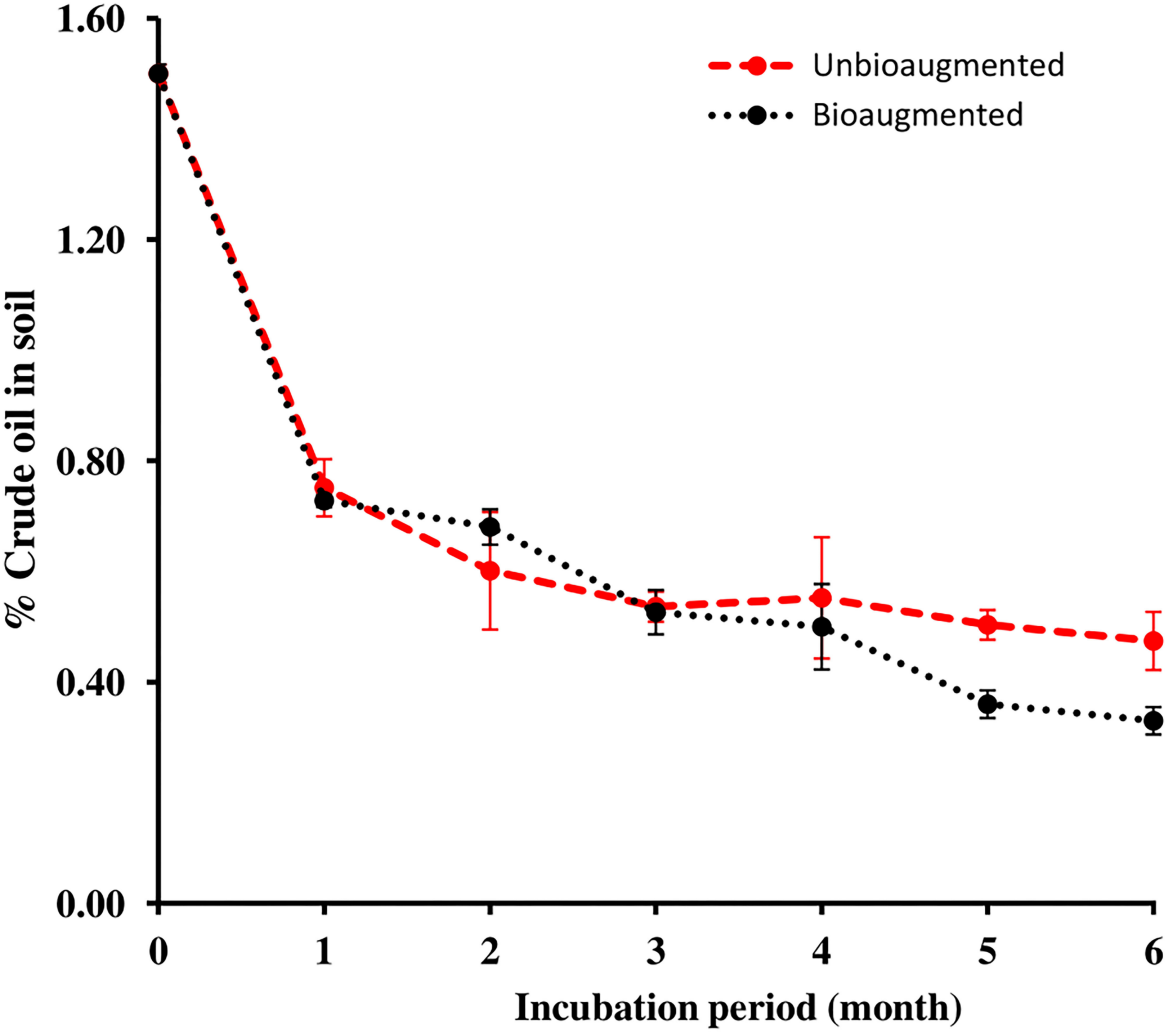
Oil removal in unbioaugmented and bioaugmented oil-polluted desert soil.

Figure 3 presents the oil removal values in the unbioaugmented and bioaugmented oil-polluted desert soil samples. Almost half of the 22.5 g (1.5 %, w/w) crude oil in each of the unbioaugmented and bioaugmented desert soils had been lost during the 1st month of the bioremediation experiment. By the end of the 6^th^ month bioremediation experiment, only 7.1 g of oil remained unconsumed in the unbioaugmented desert soil, which corresponds to a final oil removal of 68.5%. The amount of oil that remained in the bioaugmented desert soil was 5.0 g, corresponding to 74.6% of oil removal. The independent *t*-test analysis revealed that the oil removal values were significant within both unbioaugmented and the bioaugmented oil-polluted soil samples (*n* = 3, *p* < 0.05). However, there were no significant differences in the oil removal values between the unbioaugmented and the bioaugmented oil-polluted soil samples (*n* = 3, *p* > 0.05).

### Bioremediation of super oil-saturated soils

#### Number of hydrocarbonoclastic bacteria

Table 2 presents the numbers of hydrocarbonoclastic microorganisms in the desert soils polluted with different oil concentrations at time zero and after 6 months. The number of CFU in all soil samples increased from hundreds of thousands per gram of soil samples at time zero to millions and tens of millions per gram of soil samples after 6 months. The highest increase in numbers occurred in the control (oil-free) soil sample, reaching 919 × 10^5^ g^−1^. The CFU numbers in the polluted soil samples decreased as the oil concentration in the soil increased, where the CFU numbers reached 456.7 × 10^5^ g^−1^ in 1% oil concentration and reached 51.7 × 10^5^ g^−1^ in 30% oil concentration after 6 months.

**Table 2:** Number of hydrocarbonoclastic microorganisms in each oil concentration.

**Incubation**	**Numbers of CFU g**^−1^ **(x 10**^5^**)**				
**Period**	**Oil concentration (%, w/w)**				
**(months)**					
	**0**	**1**	**10**	**20**	**30**
0	2.8 ± 0.0	2.6 ± 0.0	2.7 ± 0.05	2.3± 0.0	2.5 ± 0.1
6	919.0 ± 21.0	456.7 ± 17.4	366.0 ± 13.1	130.0 ± 10.0	51.7 ± 4.6

± standard deviation values.

#### Hydrocarbonoclastic bacterial communities

**Figure 4:**
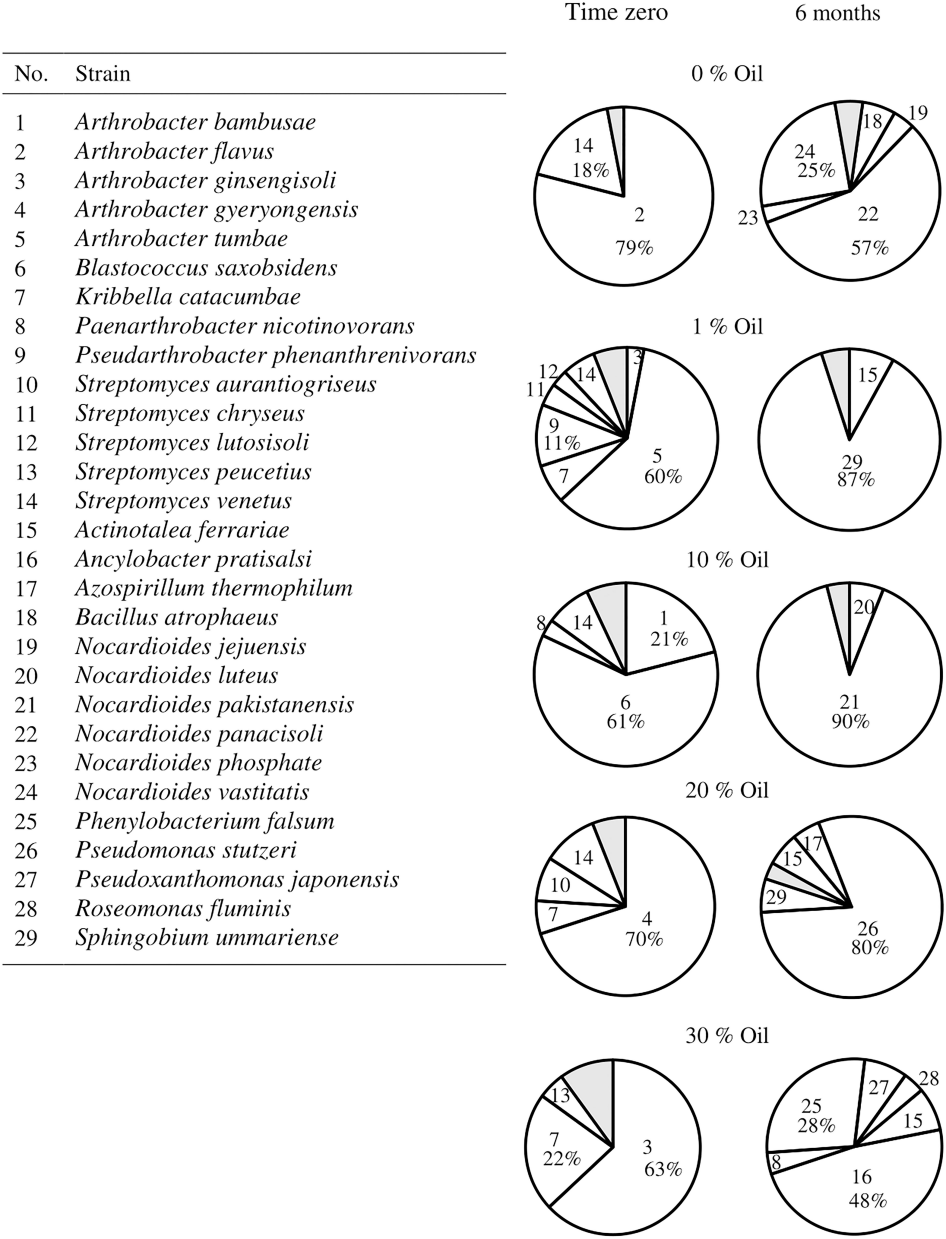
Hydrocarbonoclastic microbial communities in the desert soil samples polluted with different concentrations of crude oil at time zero and after 6 months. Shaded areas comprise minor organisms (< 5% occurrence), refer to Supplementary Table S4 for their identification.

The composition of the hydrocarbonoclastic microbial communities in all soil samples at time zero and after 6 months is presented in Figure 4. In the control (oil-free) soil samples, *Arthrobacter flavus* (79%) prevailed at time zero, whereas *Nocardioides panacisoli* (57%) prevailed after 6 months. At time zero, the most predominant strains recorded in the oil-containing soil samples were *Ar. tumbae* (60%) in 1% oil, *Blastococcus saxobsidens* (61%) in 10% oil, *Arthrobacter gyeryongensis* (70%) in 20% oil, and *Ar. ginsengisoli* (63%) in 30% oil. After 6 months, the most predominant strains were *Sphingobium ummariense* (87%) in 1% oil, *Nocardioides pakistanensis* (90%) in 10% oil, *P. stutzeri* (80%) in 20% oil, and *Ancylobacter pratisalsi* (48%) in 30% oil. Supplementary Table S2 contains the identification of the most predominant hydrocarbonoclastic bacteria isolated from each soil sample, at time zero and after 6 months, based on their 16S-rRNA gene sequences. The identified taxa had 99–100% similarity to their GenBank counterparts. The sequences were deposited in the GenBank under the accession numbers ON514452 - ON514513.

#### Oil removal by hydrocarbonoclastic bacterial communities

**Figure 5:**
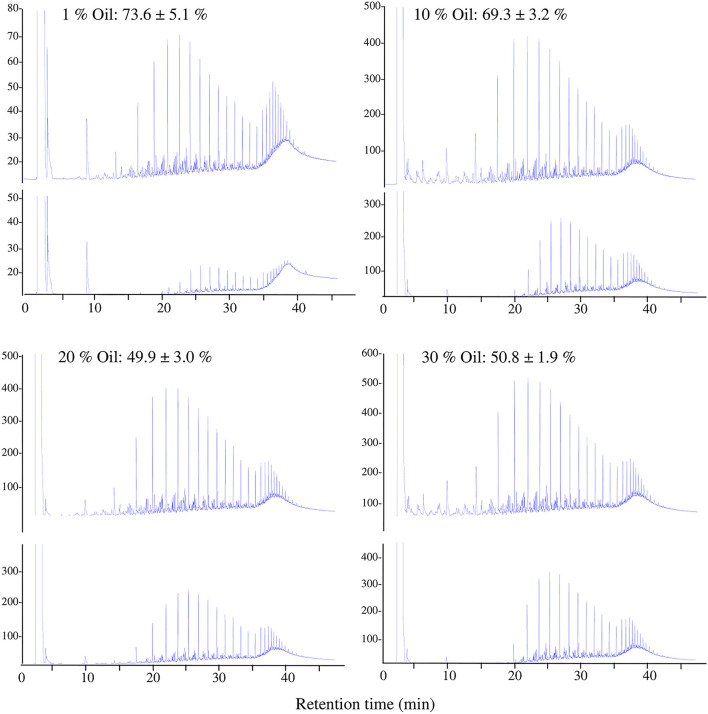
Typical GLC profiles of residual crude oil in desert soil samples polluted with different concentrations of oil at time zero (upper profiles) and after 6 months (lower profiles). Values = Oil consumed after 6 months of incubation ± standard deviation.

The GLC profiles in Figure 5 show the reduction in the oil concentration in all soil samples after 6 months of incubation. Oil-consumption rates were as high as 73.6% and 69.3% in the desert soils polluted with 1 and 10% (w/v) oil concentrations, respectively, and reached 50% in desert soils polluted with 20 and 30% (w/v) oil concentrations.

### Oil tolerance by pure isolates

**Figure 6:**
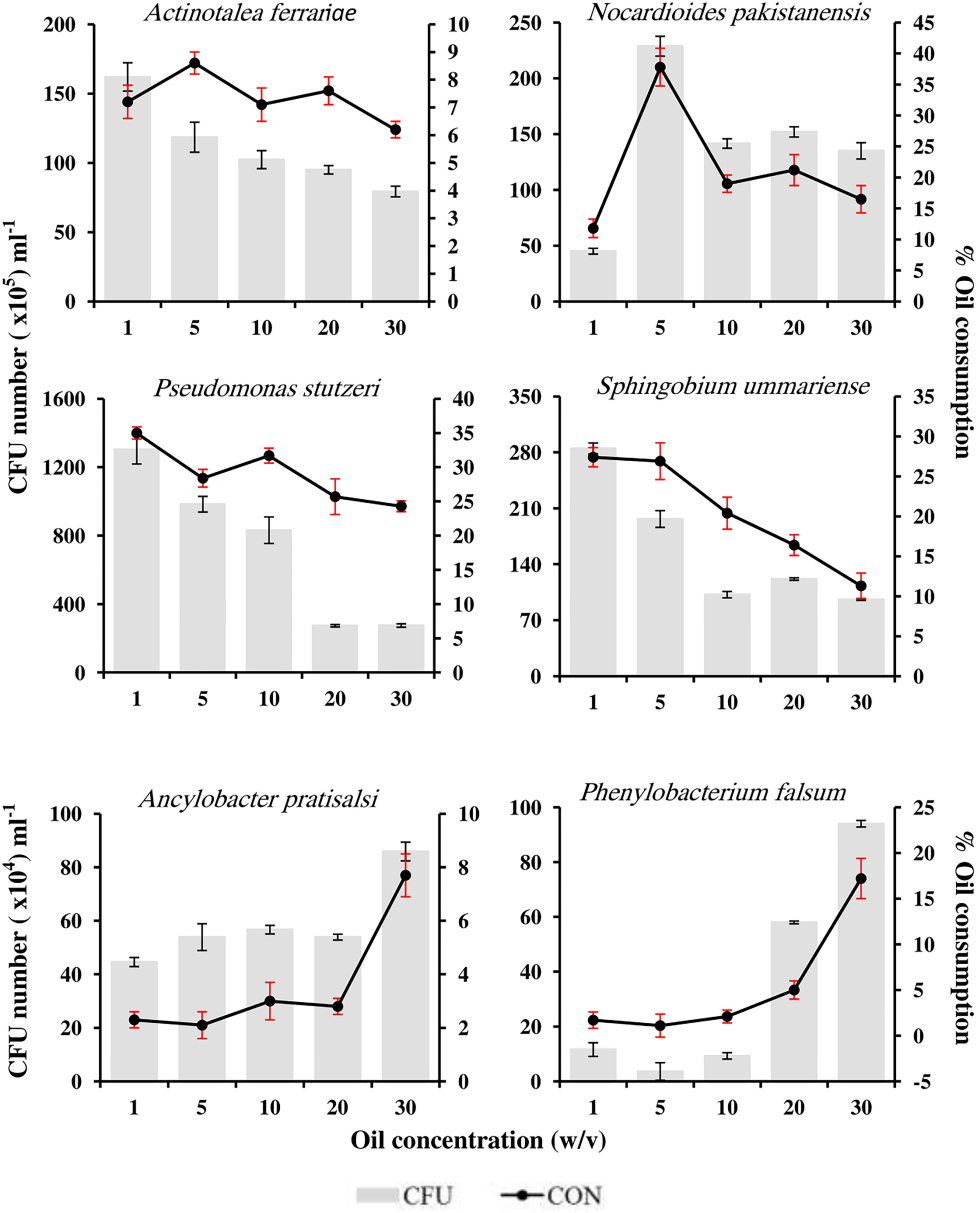
Oil tolerance of six hydrocarbonoclastic isolates.

Figure 6 shows the tolerance and the oil-consumption potential of the six tested pure isolates to different concentrations of oil in the medium (1–30 %, w/v). All the tested strains were able to grow and consume considerable proportions of the available crude oil in media containing high concentrations of oil reaching 30% (w/v). The number of CFUs was directly proportional to the oil-consumption potential. The number of four of the six tested isolates, namely, *A. ferrariae, N. pakistanensis, P. stutzeri*, and *S. ummariense*, decreased as the concentration of the oil in the medium increased; however, these isolates exhibited more oil tolerance. Among these isolates, *P. stutzeri* showed the highest tolerance to all oil concentrations, and its number reached 130 × 10^6^ CFU mL^−1^ in the medium with the initial concentration of 1% (w/v) oil and was able to consume 35% of the oil therein. The numbers decreased as the oil concentration in the medium increased; it reached 27.5 × 10^6^ CFU mL^−1^ in the medium with the initial concentration of 30% (w/v) accompanied by the consumption of 24.3% of the oil therein. Although the remaining two genera,

i.e., *An. pratisalsi* and *Phenylobacterium falsum* showed less oil tolerance, their growth was enhanced as the concentration of oil in the medium increased. The CFU numbers of *An. pratisalsi* increased from 44.6 × 10^4^ CFU mL^−1^ with oil consumption reaching 2.3% in the medium with the initial concentration of 1% (w/v) oil to 85.9 × 10^4^ CFU mL^−1^ with oil consumption reaching 7.7% in the medium with the initial concentration of 30% (w/v) oil. The number of CFU of *Ph. falsum* increased from 11.6 × 10^4^ in CFU mL^−1^ in the medium with the initial concentration of 1% (w/v) oil to 94 × 10^4^ CFU mL^−1^ in the medium with the initial concentration of 30% (w/v) oil. Consequently, the oil consumption increased from 1.7 to 17.2% in the same concentrations, respectively.

## Discussion

The results of the current study showed that, in batch culture, the consortium did not show more oil consumption than the individual species with the maximum oil consumption, i.e., *D. papillomatosis*. *P. songnenensis* took over the absolute predominance in the 5% (w/v) oil-containing mineral medium despite the fact that, among the five members of the consortium, it individually removed the least amount of oil from the mineral medium containing the same concentration of oil. *P. songnenensis* was able to overcome the stress caused by the presence of a relatively high oil concentration (5%, w/v) in the medium and even propagate therein. In the batch culture, where the nutrients are limited, *Pseudomonas* which is known to have the ability to develop advanced mechanisms to interact with other bacteria (Tashiro et al., 2013) probably succeeded to inhibit the growth of the other members of the consortium. Increasing the concentration of oil in the medium to 10% (w/v) restrained the growth of *P. songnenensis* and allowed other more tolerant members, *Ar. ginsengisoli* and *D. papillomatosis*, of the consortium to show higher CFU numbers. Apparently, the latter species were more capable of growing and propagating in higher oil concentrations (10%, w/v) in the medium than *P. songnenensis*. The absence of *A. ferrariae* and *D. cinnamea* is probably related to their low growth rates under such conditions or could be inhibited due to competition for nutrients.

Oil bioremediation by bioaugmentation with microbial consortia had been discussed extensively in many earlier published studies. Some studies reported the success of this technique in oil removal (Festa et al., 2016; Pacwa-Płociniczak et al., 2016; Brzeszcz et al., 2020), while others reported its failure (Mao et al., 2012; Al-Mailem et al., 2019; Radwan et al., 2019). The data in Table 1 show that the numbers of the hydrocarbonoclastic bacteria at time zero in the bioaugmented soil were double of those in the unbioaugmented one, mainly due to the immediate inoculation of the microbial consortium. The numbers then decreased drastically after 1 month, probably due to abiotic factors like temperature, pH, water content, and nutrient content, or biotic factors like the competition with the indigenous microorganisms for the available nutrients, which have adverse effects on the survival of the inoculated bacteria (Mrozik and Piotrowska-Seget, 2010; Varjani and Upasani, 2019). The numbers showed an increase in April and May when the outdoor temperatures were moderate and ranged between 26 and 48°C (min-max), proposing that mesophilic bacteria were the active oil degraders. The numbers in both soil samples, unbioaugmented and bioaugmented, decreased, reaching 39.7, 4.3 × 10^5^ and 42.9 × 10^5^, respectively, after 6 months of the bioremediation process, probably due to factors like limited nutrients and accumulation of toxic metabolites (Effendi and Aminati, 2019). Within this context, Hou et al. (2018) reported that some metabolic intermediates resulting from the biodegradation of petroleum hydrocarbons might show higher solubility and higher cytotoxicity to bacteria than the parent hydrocarbon. The elevated temperatures during June and July, reaching 51 °C, suggest that the active oil degraders in the studied soil samples were thermophilic/thermotolerant bacteria.

Apparently, the efficiency of bioaugmentation in oil removal depends on the ability of the consortium microorganisms to survive and remain active after inoculation (Ramos et al., 1991; Alexander, 1999; Mrozik et al., 2011; Płociniczak et al., 2013). Three of the five strains composing the consortium were detected in the soil samples taken from the bioaugmented soil immediately after inoculation (time zero), namely, *Ar. ginsengisoli, D. papillomatosis*, and *P. songnenensis*. *A. ferrariae* and *D. cinnamea* were not again detected in the collected soil samples. *Ar. ginsengisoli* and *P. songnenensis* were indigenous in the soil, since they were also detected in the unbioaugmented soil samples. Figure 2 shows no major differences between the composition of the microbial communities in the unbioaugmented and bioaugmented soil samples. In almost all the monthly collected samples during the bioremediation experiment, the taxa that predominated in the unbioaugmented soil predominated in the bioaugmented soil also. In both soil samples, *P. songnenensis* prevailed after 1 month of incubation, *S. naphthae* was one of the prevailing taxa after 3 months of incubation, and *Arthrobacter* spp prevailed through the last 3 months. *P. songnenensis*, a component of the microbial consortium, appeared in all the samples collected from the bioaugmented soil till the fourth month of incubation, proposing that it survived the environmental conditions and probably played a major role in oil bioremediation. After 4 months of incubation, indigenous species belonging to the genera *Arthrobacter* spp. (especially *Ar*. *ginsengisoli*) became the major contributor to oil bioremediation in both soils. *Ar. ginsengisoli* took over the predominance in the last 2 months with almost equal CFU counts in both soil types when the environmental parameters enhanced its growth. The shift in the microbial composition of the oil-polluted soil is understandable since oil pollution has selective effects on the soil microorganisms; after oil pollution, microorganisms with the ability to degrading the labile and less toxic hydrocarbon fractions will become active and propagate therein. After the consumption of those fractions, other microorganisms with the ability to degrade the remaining more toxic hydrocarbon fractions will predominate (Delille et al., 2003; Kaplan and Kitts, 2004).

Oil consumption values presented in Figure 3 show that the initial oil concentration in both unbioaugmented and bioaugmented soils reduced to half after 1 month of incubation. mainly due to the volatilization of low molecular weight oil hydrocarbons and microbial biodegradation of high molecular weight alkanes. At the end of the bioremediation experiment, 68.5% of the oil in the unbioaugmented soil and 74.6% of the oil in the bioaugmented were consumed, thus the bioaugmentation with the microbial consortium did not enhance the oil biodegradation in the soil dramatically, even though one of the consortium components, *P. songnenensis*, survived in the soil for 4 months. The obtained results prove that the oil in the soil was mainly degraded by indigenous bacteria to obtain the carbon and energy required for growth and reproduction and to relieve the stress caused by the presence of oil hydrocarbons in the surrounding environment (Kleindienst et al., 2015; Xu et al., 2018).

The results in Figure 5 show that the oil biodegradation process still occurred in soils polluted with high oil concentrations exceeding saturation (17.3%, w/w). The number of indigenous bacteria increased after 6 months of incubation; however, the numbers decreased as the oil concentration in the soil increased. Different indigenous bacteria successfully removed around 70% of the oil from soils contaminated with 1 and 10% oil and around 50% of the oil from those contaminated with 20 and 30% oil, defeating the harsh environmental conditions of reduced soil moisture resulting from increased soil hydrophobicity and reduced aeration due to the clogging of pore spaces by the crude oil (Klamerus-Iwan et al., 2015; Hewelke et al., 2018).

The addition of oil to the pristine soil (zero time) caused an obvious change in the bacterial structure of the soil (Figure 4 and Supplementary Table S4). The number of oil-degrading bacteria in pristine soils is normally low; once polluted, the numbers of these bacteria show a rapid increase. More than 10 species were isolated from the soil samples polluted with different oil concentrations (1–30 %, w/w), whereas only four species were isolated from the pristine one. The oil increased the hydrophobicity of the soil and released less dominant bacterial species with hydrophobic cell surfaces from the less hydrophobic soil core. An example is *Kribbella catacumbae*, which was isolated from the soil samples containing up to 30% (w/v) oil but not from the pristine one. However, different species of *Arthorbacter* and *Streptomyces* were isolated from the pristine soil samples, as well as from the oil samples after the immediate addition of oil (time zero), where hydrocarbonoclastic bacteria are known to have the ability to increase their cell surface hydrophobicity to enhance their attachment to the hydrophobic substrate (oil) to access and metabolize it (Kaczorek et al., 2013; Konieczna et al., 2018). The oil-degrading bacteria possess oxygenases, which catalyze the splitting of oxygen molecules into atoms and subsequently introduce these atoms into hydrocarbons. The attacked hydrocarbons are finally converted into fatty acids, which are further degraded by β-oxidation into acetyl-CoA units. The latter is further metabolized into cell material and energy (Rosenberg, 2006; Das and Chandran, 2011; Sui et al., 2021). Different oil-degrading bacteria possess different catalytic enzymes, and none have been shown to degrade all the constituents of crude oil. A single species has the potential to degrade a limited range of compounds.

None of the strains prevailing at time zero showed up after 6 months of incubation. New strains took over the predominance, and the composition of the microbial communities in all soil samples changed comprehensively and became less diverse. The hydrocarbon susceptibility to microbial attack depends on its molecular weight; the lower the molecular weight, the more the hydrocarbon bioavailable to the microbes. After the immediate introduction of crude oil in the soil (time zero), less toxic low molecular weight hydrocarbon fractions, i.e., *n*-alkanes and low molecular weight aromatics, would be degraded first. In this study, *Ar. tumbae* (1% oil, w/w), *B. saxobsidens* (10% oil, w/w), *Ar. ginsengisoli* (20% oil, w/w), and *Ar. ginsengisoli* (30% oil, w/w) predominated at time zero, which suggests that they were the main degraders. After the consumption of these hydrocarbon fractions, other bacteria with more advanced metabolic mechanisms capable of degrading the accumulating more toxic fractions will predominate. In this study, *S. ummariense* (1% oil, w/w), *N. pakistanensis* (10% oil, w/w), *P. stutzeri* (20% oil, w/w), *An. Pratisalsi*, and *Ph. falsum* (30% oil, w/w) took over the predominance after 6 months of incubation, suggesting that they were the major contributors to oil bioremediation and that these strains succeeded to develop mechanisms in order to survive in such a toxic environment. In this context, a published study by Goudarztalejerdi et al. (2015) reported that several *Pseudomonas* strains isolated from oil-polluted soils have the ability to produce polyhydroxyalkanoic acid (PHA) using crude oil as a substrate, which might indicate a possible role of PHA in bacterial survival in the stressed environment. However, this study also reported the failure of these strains to grow in liquid cultures containing more than 2% (v/v) crude oil. Although the number of oil-degrading bacteria increased in all oil-polluted soil samples (1–30%, w/w) after 6 months of incubation, the number of species declined from 10–11 species at time zero to 5–6 species after 6 months in all of them. High oil concentrations have toxic effects on oil-degrading bacteria, reducing their activity and causing their death, thus reducing the stability of the community (Wang et al., 2021).

The ability of the six tested isolates to assimilate high concentrations of oil in liquid medium (1–30%, w/v) (Figure 6) is expected due to their adaptation as a consequence of their previous exposure to oil in the polluted soil. The ability of the isolates to live and propagate in such high oil concentrations may indicate their ability to emulsify oil hydrocarbons to facilitate their uptake. Although the mechanism(s) developed to overcome the toxic effects of such high oil concentrations in the medium merit more studies and experimentation, it is beyond the scope of this study. The effectiveness of oil biodegradation is normally affected by many external factors, like temperature, pH, and oxygen. To recall, the culture flasks contained an inorganic medium (pH 7.0) with oil as the sole source of carbon and energy, and the cultures were incubated at 30 °C with continuous shaking. These conditions were considered ideal for oil biodegradation (Das and Chandran, 2011). Oil degradation rates increased as the number of the tested bacteria in the liquid medium increased. Increasing the concentration of oil in the liquid medium increased oil toxicity on the cells and reduced bacterial numbers, except for *An. pratisalsi* and *Ph. Falsum*, whose numbers and oil biodegradation rates remarkably increased with increasing oil concentrations in the liquid medium.

## Conclusion

The presented work studies the feasibility of using a consortium of five hydrocarbonoclastic bacterial strains (*A. ferrariae, Ar. ginsengisoli, D. papillomatosis, D. cinnamea*, and *P. songnenensis*) with high oil degradation potential for enhancing bioremediation. The consortium failed, in batch culture, to bring about more oil consumption than the individual strain with the maximum oil consumption, i.e., *D. papillomatosis*. This study showed that the oil degradation potential of a consortium is not necessarily the total sum of the capacities of the individual members of the consortium. The consortium failed to enhance bioremediation in soil microcosms too. The metabolic activities of the oil-degrading bacteria are not only affected by their own activities but also affected by the surrounding environmental factors too. One of the most difficult issues facing the added strains is to survive in the soil defeating the abiotic and biotic stresses therein. Of the five consortium members, only *Ar. ginsengisoli* and *P. songnenensis* were isolated from some of the collected bioaugmented soil samples during the bioremediation process. However, these two strains were also isolated from some of the collected unbioaugmented soil samples during the bioremediation process; apparently, they were indigenous in the soil.

On the other hand, indigenous soil bacteria, when exposed to oil, have the potential to adapt and develop various mechanisms necessary to utilize the surrounding oil as a source of carbon and energy in a relatively short span of time and exhibit high oil degradation rates. Oil pollution usually changes the community structure, resulting in the development of dominant microbial communities with enhanced physiological and oil utilization capabilities that can survive the toxic effects of pollution. Our work showed that even in high oil concentrations, reaching 30% w/w, the soils were enriched with bacterial strains capable of tolerating and degrading such high concentrations of oil and propagating therein.

In conclusion, this study showed that bioaugmenting oil-polluted soil samples with a microbial consortium of hydrocarbonoclastic bacterial species with high oil removal potential did not drastically enhance oil bioremediation or affected its effective hydrocarbonoclastic bacterial makeup. In oil-polluted soils, self-cleaning proceeds through the enrichment of minor indigenous bacteria having the ability to degrade oil and propagate therein. This study proved that this was true even in super oil-saturated soils.

## Data availability statement

The datasets presented in this study can be found in online repositories. The names of the repository/repositories and accession number(s) can be found in the article/Supplementary material.

## Author contributions

HA-A suggested the research topic. HA-A, NA, and MK contributed to the experimental design. MK performed the experimental work. All authors contributed to the manuscript.

## Funding

This work was supported by Kuwait University, Research Grant RS 01/19.

## Conflict of interest

The authors declare that the research was conducted in the absence of any commercial or financial relationships that could be construed as a potential conflict of interest.

## Publisher's note

All claims expressed in this article are solely those of the authors and do not necessarily represent those of their affiliated organizations, or those of the publisher, the editors and the reviewers. Any product that may be evaluated in this article, or claim that may be made by its manufacturer, is not guaranteed or endorsed by the publisher.
