# Supplementary material for: Indigenous oil-degrading bacteria more efficient in soil bioremediation than microbial consortium and active even in super oil-saturated soils
Source: Front Microbiol. 2022 Aug 1;13:950051. doi: 10.3389/fmicb.2022.950051 (PMC9376284; doi:10.3389/fmicb.2022.950051)
Supplement: Supplementary file 1 [file Data_Sheet_1.docx]

**Table S1** Sequencing of 16S rRNA-genes of hydrocarbonoclastic bacteria isolated from unbioaugmented and bioaugmented oil-polluted desert soils.

| Isolate | Total bases | Class | Nearest Gene Bank match | Similarity % | Bases compared | Accession number |
| --- | --- | --- | --- | --- | --- | --- |
| 1 | 418 | Actinobacteria | *Actinotalea solisilvae* (NR_159882) | 100 | 418/418 | MN905421 |
| 2 | 490 | Actinobacteria | *Amycolatopsis oliviviridis* (LC225637) | 100 | 490/490 | MN905405 |
| 3 | 486 | Actinobacteria | *Arthrobacter crystallopoietes* (NR_026189) | 100 | 486/486 | MN905381 |
| 4 | 492 | Actinobacteria | *Arthrobacter echini* ([NR_148833)](https://www.ncbi.nlm.nih.gov/nucleotide/NR_148833.1?report=genbank&log$=nuclalign&blast_rank=1&RID=ASS2U0G1015) | 99 | 495/498 | MN905377 |
| 5 | 502 | Actinobacteria | *Arthrobacter ginsengisoli* ([KF212463)](https://www.ncbi.nlm.nih.gov/nucleotide/KF212463.1?report=genbank&log$=nuclalign&blast_rank=2&RID=ASTFHW15014) | 100 | 502/502 | MN905388 |
| 6 | 489 | Actinobacteria | *Arthrobacter tumbae* ([NR_042078](https://www.ncbi.nlm.nih.gov/nucleotide/NR_042078.1?report=genbank&log$=nuclalign&blast_rank=1&RID=ASTFHW15014)) | 100 | 489/489 | MN905385 |
| 7 | 479 | α-Proteobacteria | *Azospirillum rugosum* ([NR_042582](https://www.ncbi.nlm.nih.gov/nucleotide/NR_042582.1?report=genbank&log$=nuclalign&blast_rank=1&RID=15HEV7EF014)) | 99 | 493/500 | MN905423 |
| 8 | 456 | α-Proteobacteria | *Belnapia soli* ([NR_109456](https://www.ncbi.nlm.nih.gov/nucleotide/NR_109456.1?report=genbank&log$=nuclalign&blast_rank=1&RID=ASS2U0G1015)) | 99 | 462/465 | MN905376 |
| 9 | 488 | α-Proteobacteria | *Carbophilus carboxidus* (NR_104931) | 98 | 508/518 | MN905403 |
| 10 | 517 | Actinobacteria | *Cellulomonas iranensis* (NR_024914) | 99 | 525/529 | MN905425 |
| 11 | 523 | Actinobacteria | *Cellulomonas pakistanensis* (NR_125452) | 99 | 525/526 | MN905410 |
| 12 | 523 | Actinobacteria | *Cellulosimicrobium funkei* ([NR_042937](https://www.ncbi.nlm.nih.gov/nucleotide/NR_042937.1?report=genbank&log$=nuclalign&blast_rank=1&RID=0KDG48P9014)) | 100 | 523/523 | MN905424 |
| 13 | 488 | Actinobacteria | *Dietzia papillomatosis* ([NR_116687](https://www.ncbi.nlm.nih.gov/nucleotide/NR_116687.1?report=genbank&log$=nuclalign&blast_rank=1&RID=ASWRUP5C014)) | 100 | 488/488 | MN905387 |
| 14 | 501 | α-Proteobacteria | *Enterovirga rhinocerotis* (NR_149206) | 99 | 511/516 | MN905434 |
| 15 | 514 | γ-Proteobacteria | *Escherichia coli* ([LC069032](https://www.ncbi.nlm.nih.gov/nucleotide/LC069032.1?report=genbank&log$=nuclalign&blast_rank=6&RID=ASTFHW15014)) | 100 | 514/514 | MN905389 |
| 16 | 511 | Actinobacteria | *Gordonia didemni* ([NR_146040](https://www.ncbi.nlm.nih.gov/nucleotide/NR_146040.1?report=genbank&log$=nuclalign&blast_rank=1&RID=EZ5YXDKF015)) | 99 | 521/526 | MN905402 |
| 17 | 512 | Actinobacteria | *Gordonia hankookensis* (NR_104507) | 99 | 518/521 | MN905411 |
| 18 | 491 | Actinobacteria | *Gordonia hongkongensis* (NR_152022) | 99 | 493/494 | MN905394 |
| 19 | 492 | Actinobacteria | *Janibacter hoylei* ([NR_104794)](https://www.ncbi.nlm.nih.gov/nucleotide/NR_104794.1?report=genbank&log$=nuclalign&blast_rank=1&RID=ASTFHW15014) | 99 | 496/498 | MN905391 |
| 20 | 523 | Actinobacteria | *Kocuria flava* (NR_044308) | 99 | 527/529 | MN905432 |
| 21 | 496 | Actinobacteria | *Kocuria polaris* (KX959605) | 100 | 496/496 | MN905400 |
| 22 | 515 | β-Proteobacteria | *Massilia agri* (NR_157781) | 99 | 521/524 | MN905435 |
| 23 | 508 | β-Proteobacteria | *Massilia lutea* ([NR_043310)](https://www.ncbi.nlm.nih.gov/nucleotide/NR_043310.1?report=genbank&log$=nuclalign&blast_rank=1&RID=ASWWXZDK015) | 99 | 510/511 | MN905390 |
| 24 | 481 | α-Proteobacteria | *Mesorhizobium carbonis* (NR_164961) | 99 | 485/487 | MN905399 |
| 25 | 416 | α-Proteobacteria | *Methylobacterium gregans* (NR_041440) | 99 | 418/419 | MN905395 |
| 26 | 513 | α-Proteobacteria | *Methylobacterium phyllostachyos* (NR_108242) | 100 | 513/513 | MN905441 |
| 27 | 514 | α-Proteobacteria | *Methylorubrum podarium* ([NR_112676](https://www.ncbi.nlm.nih.gov/nucleotide/NR_112676.1?report=genbank&log$=nuclalign&blast_rank=1&RID=ASS2U0G1015)) | 100 | 514/514 | MN905442 |
| 28 | 481 | α-Proteobacteria | *Methylorubrum populi* ([NR_074257)](https://www.ncbi.nlm.nih.gov/nucleotide/NR_074257.1?report=genbank&log$=nuclalign&blast_rank=2&RID=ASS2U0G1015) | 100 | 481/481 | MN905378 |
| 29 | 503 | α-Proteobacteria | *Methylorubrum rhodinum* (NR_041029) | 99 | 513/518 | MN905401 |
| 30 | 512 | Actinobacteria | *Microbacterium aureliae* ([NR_152652)](https://www.ncbi.nlm.nih.gov/nucleotide/NR_152652.1?report=genbank&log$=nuclalign&blast_rank=1&RID=0P2H98KH016) | 100 | 512/512 | MN905447 |
| 31 | 493 | Actinobacteria | *Microbacterium lacusdiani* ([KP986565](https://www.ncbi.nlm.nih.gov/nucleotide/KP986565.1?report=genbank&log$=nuclalign&blast_rank=2&RID=ASS2U0G1015)) | 99 | 495/496 | MN905380 |
| 32 | 484 | Actinobacteria | *Microbacterium oryzae* (NR_117527) | 99 | 490/493 | MN905408 |
| 33 | 494 | Actinobacteria | *Microbacterium paraoxydans* (MH281749) | 99 | 496/497 | MN905407 |
| 34 | 518 | Actinobacteria | *Mycobacterium anyangense* (KF910200) | 99 | 520/521 | MN905430 |
| 35 | 512 | Actinobacteria | *Mycobacterium arabiense* (KC010491) | 99 | 514/515 | MN905448 |
| 36 | 517 | Actinobacteria | *Mycobacterium cosmeticum* (MH169226) | 99 | 521/523 | MN905409 |
| 37 | 512 | Actinobacteria | *Mycobacterium farcinogenes* ([LT718447](https://www.ncbi.nlm.nih.gov/nucleotide/LT718447.1?report=genbank&log$=nuclalign&blast_rank=2&RID=0NY6DFWT014)) | 99 | 514/515 | MN905446 |
| 38 | 514 | Actinobacteria | *Mycobacterium hackensackense* ([NR_115184](https://www.ncbi.nlm.nih.gov/nucleotide/NR_115184.1?report=genbank&log$=nuclalign&blast_rank=1&RID=0NXKVGB8014)) | 100 | 514/514 | MN905445 |
| 39 | 513 | Actinobacteria | *Mycolicibacterium iranicum* (NR_117909) | 99 | 515/516 | MN905440 |
| 40 | 512 | Actinobacteria | *Mycolicibacterium litorale* (NR_117568) | 99 | 514/515 | MN905444 |
| 41 | 513 | Actinobacteria | *Mycolicibacterium murale* (NR_117884) | 100 | 513/513 | MN905420 |
| 42 | 505 | Actinobacteria | *Nocardia cyriacigeorgica* ([NR_117334)](https://www.ncbi.nlm.nih.gov/nucleotide/NR_117334.1?report=genbank&log$=nuclalign&blast_rank=1&RID=ASTFHW15014) | 99 | 511/514 | MN905443 |
| 43 | 516 | Actinobacteria | *Nocardia fluminea* ([NR_117325)](https://www.ncbi.nlm.nih.gov/nucleotide/NR_117325.1?report=genbank&log$=nuclalign&blast_rank=1&RID=0NU5AKZB016) | 100 | 516/516 | MN905451 |
| 44 | 510 | Actinobacteria | *Nocardia rhizosphaerihabitans* (NR_159262) | 99 | 516/519 | MN905404 |
| 45 | 515 | Actinobacteria | *Nocardia takedensis* (NR_118210) | 100 | 515/515 | MN905452 |
| 46 | 510 | Actinobacteria | *Nocardia testacea* (NR_118212) | 99 | 518/522 | MN905417 |
| 47 | 501 | Actinobacteria | *Nocardioides aromaticivorans* (T) ([MK424297](https://www.ncbi.nlm.nih.gov/nucleotide/MK424297.1?report=genbank&log$=nuclalign&blast_rank=1&RID=0KBPSED101R)) | 100 | 501/501 | MN905413 |
| 48 | 480 | Actinobacteria | *Nocardioides cavernae* ([NR_156135](https://www.ncbi.nlm.nih.gov/nucleotide/NR_156135.1?report=genbank&log$=nuclalign&blast_rank=1&RID=ASTFHW15014)) | 99 | 484/486 | MN905386 |
| 49 | 514 | Actinobacteria | *Nocardioides flavus* (AF005014) | 100 | 514/514 | MN905419 |
| 50 | 490 | Actinobacteria | *Nocardioides marinisabuli* ([NR_042590](https://www.ncbi.nlm.nih.gov/nucleotide/NR_042590.1?report=genbank&log$=nuclalign&blast_rank=1&RID=0KEA8YVX016)) | 99 | 497/501 | MN905433 |
| 51 | 524 | β-Proteobacteria | *Noviherbaspirillum canariense* (NR_118039) | 99 | 529/530 | MN905412 |
| 52 | 497 | β-Proteobacteria | *Noviherbaspirillum denitrificans* (NR_157007) | 98 | 515/524 | MN905436 |
| 53 | 523 | β-Proteobacteria | *Noviherbaspirillum soli* (NR_118041) | 99 | 524/525 | MN905431 |
| 54 | 499 | Bacilli | *Paenibacillus lautus* (NR_117185) | 99 | 501/502 | MN905396 |
| 55 | 501 | γ-Proteobacteria | *Pantoea agglomerans* (NR_041978) | 99 | 505/507 | MN905379 |
| 56 | 532 | γ-Proteobacteria | *Pantoea brenneri* ([NR_116748](https://www.ncbi.nlm.nih.gov/nucleotide/NR_116748.1?report=genbank&log$=nuclalign&blast_rank=1&RID=ASS2U0G1015)) | 99 | 533/534 | MN905382 |
| 57 | 493 | Actinobacteria | *Pseudarthrobacter phenanthrenivorans* (NR_042469) | 100 | 493/493 | MN905398 |
| 58 | 518 | γ-Proteobacteria | *Pseudomonas songnenensis* ([NR_148295)](https://www.ncbi.nlm.nih.gov/nucleotide/NR_148295.1?report=genbank&log$=nuclalign&blast_rank=1&RID=ASTFHW15014) | 99 | 520/521 | MN905392 |
| 59 | 537 | γ-Proteobacteria | *Pseudoxanthomonas japonensis* ([NR_113972](https://www.ncbi.nlm.nih.gov/nucleotide/NR_113972.1?report=genbank&log$=nuclalign&blast_rank=1&RID=0KC4HMZ501R)) | 99 | 541/545 | MN905415 |
| 60 | 512 | Actinobacteria | *Rhodococcus cercidiphylli* (LC130641) | 99 | 514/515 | MN905427 |
| 61 | 513 | Actinobacteria | *Rhodococcus pedocola* (NR_149270) | 100 | 513/513 | MN905422 |
| 62 | 487 | α-Proteobacteria | *Sinorhizobium meliloti* (T) ([MH661212](https://www.ncbi.nlm.nih.gov/nucleotide/MH661212.1?report=genbank&log$=nuclalign&blast_rank=1&RID=ASTFHW15014)) | 100 | 487/487 | MN905397 |
| 63 | 494 | α-Proteobacteria | *Skermanella aerolata* (NR_043929) | 100 | 494/494 | MN905437 |
| 64 | 490 | α-Proteobacteria | *Skermanella rosea* (NR_152076) | 99 | 500/505 | MN905414 |
| 65 | 492 | α-Proteobacteria | *Sphingobium naphthae* ([NR_157779)](https://www.ncbi.nlm.nih.gov/nucleotide/NR_157779.1?report=genbank&log$=nuclalign&blast_rank=1&RID=ASTFHW15014) | 100 | 492/492 | MN905393 |
| 66 | 516 | α-Proteobacteria | *Sphingomonas zeicaulis* (NR_152012) | 99 | 520/522 | MN905428 |
| 67 | 515 | Actinobacteria | *Streptomyces abikoensis* ([KC954556](https://www.ncbi.nlm.nih.gov/nucleotide/KC954556.1?report=genbank&log$=nuclalign&blast_rank=1&RID=0NV53DP6016)) | 100 | 515/515 | MN905438 |
| 68 | 508 | Actinobacteria | *Streptomyces alanosinicus* ([NR_117991)](https://www.ncbi.nlm.nih.gov/nucleotide/NR_117991.1?report=genbank&log$=nuclalign&blast_rank=1&RID=ASTFHW15014) | 99 | 512/514 | MN905449 |
| 69 | 515 | Actinobacteria | *Streptomyces chryseus* (MK583947) | 99 | 519/521 | MN905429 |
| 70 | 505 | Actinobacteria | *Streptomyces exfoliatus* (MG657246) | 99 | 511/514 | MN905418 |
| 71 | 479 | Actinobacteria | *Streptomyces fumanus* ([NR_041101)](https://www.ncbi.nlm.nih.gov/nucleotide/NR_041101.1?report=genbank&log$=nuclalign&blast_rank=1&RID=0NVCBVB4016) | 98 | 503/515 | MN905439 |
| 72 | 482 | Actinobacteria | *Streptomyces kalpinensis* ([NR_15919)](https://www.ncbi.nlm.nih.gov/nucleotide/NR_159194.1?report=genbank&log$=nuclalign&blast_rank=1&RID=ASTFHW15014) | 99 | 484/485 | MN905383 |
| 73 | 515 | Actinobacteria | *Streptomyces purpurascens* ([NR_042101](https://www.ncbi.nlm.nih.gov/nucleotide/NR_042101.2?report=genbank&log$=nuclalign&blast_rank=3&RID=0P4MZJ50014)) | 100 | 515/515 | MN905450 |
| 74 | 509 | Actinobacteria | *Streptomyces thermospinosisporus* (KU141346) | 99 | 517/521 | MN905416 |
| 75 | 521 | Actinobacteria | *Tsukamurella strandjordii* ([KX924563](https://www.ncbi.nlm.nih.gov/nucleotide/KX924563.1?report=genbank&log$=nuclalign&blast_rank=1&RID=ASTFHW15014)) | 99 | 522/523 | MN905384 |
| 76 | 492 | Actinobacteria | *Williamsia marianensis* ([NR_118613)](https://www.ncbi.nlm.nih.gov/nucleotide/NR_118613.1?report=genbank&log$=nuclalign&blast_rank=1&RID=ASTFHW15014) | 100 | 492/492 | MN905406 |
| 77 | 517 | α-Proteobacteria | *Xanthobacter flavus* (NR_113665) | 99 | 519/520 | MN905426 |

**TABLE S2** Sequencing of 16S rRNA-gene of hydrocarbonoclastic bacteria isolated from the desert soil samples polluted with different concentrations of crude oil at time zero and after six months.

| Isolate designation | Total bases | Subdivision | Nearest Gene Bank match | Similarity % | Bases compared | Accession number |
| --- | --- | --- | --- | --- | --- | --- |
| Time zero |  |  |  |  |  |  |
| 0M-0-2 | 515 | Actinobacteria | *Arthrobacter flavus* (KT989846) | 100 | 515/515 | ON514452 |
| 0M-0-8 | 542 | Proteobacteria | *Enterobacter sichuanensis* (MG832788) | 100 | 542/542 | ON514453 |
| 0M-0-10 | 513 | Actinobacteria | *Streptomyces venetus* (LC073310) | 99 | 515/516 | ON514454 |
| 0M-0-21 | 522 | Actinobacteria | *Micromonospora terminaliae* (NR_156056) | 100 | 522/522 | ON514455 |
| 0M-0-22 | 533 | Actinobacteria | *Streptomyces venetus* (LC073310) | 100 | 533/533 | ON514456 |
| 0M-1-29 | 532 | Actinobacteria | *Kocuria turfanensis* (MN826469) | 99 | 538/541 | ON514457 |
| 0M-1-31 | 533 | Actinobacteria | *Pseudarthrobacter phenanthrenivorans* (NR_042469) | 100 | 533/533 | ON514458 |
| 0M-1-32 | 521 | Actinobacteria | *Arthrobacter tumbae* (NR_042078) | 100 | 521/521 | ON514459 |
| 0M-1-33 | 516 | Actinobacteria | *Arthrobacter ginsengisoli* (KF212463) | 100 | 516/516 | ON514460 |
| 0M-1-46 | 531 | α-Proteobacteria | *Sinorhizobium meliloti* (MH661212) | 99 | 539/543 | ON514461 |
| 0M-1-47 | 472 | Bacilli | *Bacillus muralis* (MN326679) | 99 | 478/481 | ON514462 |
| 0M-1-52 | 520 | Actinobacteria | *Kribbella catacumbae* (NR_042657) | 99 | 526/529 | ON514463 |
| 0M-1-59 | 466 | Actinobacteria | *Streptomyces tibetensis* (NR_165779) | 100 | 466/466 | ON514464 |
| 0M-1-64 | 533 | Actinobacteria | *Streptomyces lutosisoli* (NR_164920) | 100 | 533/533 | ON514465 |
| 0M-10-65 | 534 | γ-Proteobacteria | *Pseudoxanthomonas japonensis* (NR_113972) | 99 | 540/543 | ON514466 |
| 0M-10-66 | 533 | Actinobacteria | *Paenarthrobacter nicotinovorans* (MK424298) | 100 | 533/533 | ON514467 |
| 0M-10-67 | 519 | Actinobacteria | *Arthrobacter bambusae* (NR_133968) | 99 | 521/522 | ON514468 |
| 0M-10-68 | 520 | Actinobacteria | *Paenarthrobacter nitroguajacolicus* (MK424299) | 99 | 528/532 | ON514469 |
| 0M-10-69 | 545 | γ-Proteobacteria | *Pantoea dispersa* (MT779002) | 100 | 545/545 | ON514470 |
| 0M-10-77 | 529 | Bacilli | *Paenibacillus xylanexedens* (MF347934) | 99 | 533/535 | ON514471 |
| 0M-10-80 | 534 | Actinobacteria | *Blastococcus saxobsidens* (MK318567) | 99 | 540/543 | ON514472 |
| 0M-10-95 | 512 | Actinobacteria | *Streptomyces deserti* (NR_117576) | 99 | 518/521 | ON514473 |
| 0M-20-99 | 546 | Actinobacteria | *Arthrobacter gyeryongensis* (NR_133699) | 100 | 546/546 | ON514474 |
| 0M-20-108 | 493 | Actinobacteria | *Nocardioides luteus* (MT760455) | 100 | 493/493 | ON514475 |
| 0M-20-112 | 536 | γ-Proteobacteria | *Acinetobacter johnsonii* (LT899949) | 99 | 538/539 | ON514476 |
| 0M-20-124 | 511 | Actinobacteria | *Streptomyces aurantiogriseus* (NR_041081) | 99 | 521/526 | ON514477 |
| 0M-20-130 | 457 | Actinobacteria | *Streptomyces blastmyceticus* (MT760573) | 99 | 469/475 | ON514478 |
| 0M-20-132 | 510 | Actinobacteria | *Streptomyces paludis* (MH636862) | 99 | 520/525 | ON514479 |
| 0M-30-137 | 505 | Actinobacteria | *Streptomyces chryseus* (MK583947) | 100 | 505/505 | ON514480 |
| 0M-30-143 | 522 | Actinobacteria | *Streptomyces violaceorubidus* (NR_112474) | 99 | 536/543 | ON514481 |
| 0M-30-148 | 551 | Bacilli | *Bacillus halosaccharovorans* (NR_109116) | 99 | 553/555 | ON514482 |
| 0M-30-152 | 503 | Actinobacteria | *Streptomyces peucetius* (NR_024763) | 99 | 511/515 | ON514483 |
| 0M-30-155 | 519 | Bacilli | *Bacillus fumarioli* (NR_025370) | 99 | 527/531 | ON514484 |
| 0M-30-164 | 523 | Actinobacteria | *Kribbella sandramycini* (NR_024845) | 100 | 523/523 | ON514485 |
| 0M-30-167 | 515 | Actinobacteria | *Streptomyces indoligenes* (NR_149274) | 99 | 523/527 | ON514486 |
| 0M-30-168 | 522 | Actinobacteria | *Streptomyces griseoflavus* (KU720585) | 99 | 528/531 | ON514487 |
| 6 months |  |  |  |  |  |  |
| 6M-0-1 | 531 | Actinobacteria | *Nocardioides jejuensis* (MH725561) | 99 | 541/546 | ON514488 |
| 6M-0-2 | 484 | Actinobacteria | *Nocardioides panacisoli* (NR_104528) | 99 | 492/496 | ON514489 |
| 6M-0-3 | 511 | Bacilli | *Bacillus atrophaeus* (MN840037) | 99 | 517/520 | ON514490 |
| 6M-0-5 | 506 | Actinobacteria | *Nocardioides ganghwensis* (AY423718) | 99 | 510/512 | ON514491 |
| 6M-0-7 | 540 | Actinobacteria | *Nocardioides vastitatis* (MK787305) | 99 | 546/549 | ON514492 |
| 6M-0-9 | 441 | Actinobacteria | *Nocardioides phosphati* (NR_156869) | 98 |  | ON514493 |
| 6M-0-12 | 454 | Actinobacteria | *Nocardioides taihuensis* (NR_158043) | 99 | 460/463 | ON514494 |
| 6M-1-13 | 516 | Actinobacteria | *Gordonia didemni* (NR_146040) | 100 | 516/516 | ON514495 |
| 6M-1-24 | 547 | Actinobacteria | *Pseudarthrobacter polychromogenes* (MT760382) | 99 | 549/550 | ON514496 |
| 6M-1-27 | 536 | Actinobacteria | *Streptomyces indoligenes* (NR_149274) | 100 | 536/536 | ON514497 |
| 6M-1-28 | 527 | Actinobacteria | *Streptomyces thermospinosisporus* (KU141346) | 100 | 527/527 | ON514498 |
| 6M-10-29 | 545 | Actinobacteria | *Actinotalea ferrariae* (NR_118034) | 100 | 545/545 | ON514499 |
| 6M-10-32 | 495 | Actinobacteria | *Nocardioides pakistanensis* (NR_146700) | 99 | 501/504 | ON514500 |
| 6M-10-34 | 531 | Actinobacteria | *Agromyces indicus* (NR_108908) | 99 | 541/546 | ON514501 |
| 6M-10-35 | 544 | Actinobacteria | *Arthrobacter ginsengisoli* (KF212463) | 99 | 548/550 | ON514502 |
| 6M-0-37 | 442 | Actinobacteria | *Nocardioides luteus* (MT760455) | 99 | 444/445 | ON514503 |
| 6M-20-42 | 498 | α-Proteobacteria | *Azospirillum thermophilum* (MH265951) | 99 | 504/507 | ON514504 |
| 6M-20-43 | 492 | α-Proteobacteria | *Mesorhizobium thiogangeticum* (NR_042358) | 99 | 504/510 | ON514505 |
| 6M-20-49 | 531 | γ-Proteobacteria | *Pseudomonas stutzeri* (MT027239) | 99 | 535/537 | ON514506 |
| 6M-20-50 | 508 | α-Proteobacteria | *Sphingobium ummariense* (NR_044171) | 100 | 508/508 | ON514507 |
| 6M-20-51 | 509 | Actinobacteria | *Intrasporangium calvum* (NR_074507) | 99 | 519/524 | ON514508 |
| 6M-30-54 | 502 | α-Proteobacteria | *Ancylobacter pratisalsi* (NR_159167) | 99 | 508/511 | ON514509 |
| 6M-30-59 | 506 | α-Proteobacteria | *Phenylobacterium falsum (*NR_042277) | 99 | 514/518 | ON514510 |
| 6M-30-64 | 480 | α-Proteobacteria | *Roseomonas fluminis* (NR_159916) | 99 | 492/498 | ON514511 |
| 6M-30-65 | 543 | γ-Proteobacteria | *Pseudoxanthomonas japonensis* (NR_113972) | 99 | 549/552 | ON514512 |
| 6M-30-66 | 444 | Actinobacteria | *Paenarthrobacter nicotinovorans* (MK424298) | 99 | 448/450 | ON514513 |

**Table S3.** Minor hydrocarbonoclastic bacteria in the unbioaugmented and bioaugmented oil-polluted desert soil during bioremediation (shaded areas in Pie charts in Figure 2).

| Incubation period (months) | Unbioaugmented |  | Bioaugmented |
| --- | --- | --- | --- |
| 0 | *Amycolatopsis oliviviridis* |  | *Escherichia coli* |
|  | *Arthrobacter echini* |  | *Massilia lutea* |
|  | *Belnapia soli* |  |  |
|  | *Methylorubrum populi* |  |  |
|  | *Nocardia cyriacigeorgica* |  |  |
|  | *Streptomyces kalpinensis* |  |  |
|  | *Tsukamurella strandjordii* |  |  |
| 1 | *Janibacter hoylei* |  |  |
|  | *Williamsia marianensis* |  |  |
| 2 | *Gordonia hankookensis* |  | *Kocuria polaris* |
|  | *Methylobacterium gregans* |  | *Pseudarthrobacter phenanthrenivorans* |
| 3 | *Amycolatopsis oliviviridis* |  | *Cellulomonas pakistanensis* |
|  | *Arthrobacter crystallopoietes* |  | *Methylorubrum populi* |
|  | *Gordonia didemni* |  | *Mycobacterium cosmeticum* |
|  | *Mycolicibacterium iranicum* |  | *Nocardia takedensis* |
|  | *Nocardia rhizosphaerihabitans* |  | *Sinorhizobium meliloti* |
| 4 | *Gordonia hankookensis* |  | *Arthrobacter ginsengisoli* |
|  | *Methylobacterium phyllostachyos* |  | *Azospirillum rugosum* |
|  | *Mycobacterium anyangense* |  | *Cellulomonas iranensis* |
|  | *Mycolicibacterium murale* |  | *Cellulosimicrobium funkei* |
|  | *Nocardia cyriacigeorgica* |  | *Methylobacterium phyllostachyos* |
|  | *Nocardia testacea* |  | *Methylorubrum podarium* |
|  | *Nocardioides aromaticivorans* |  | *Methylorubrum populi* |
|  | *Nocardioides cavernae* |  | *Microbacterium paraoxydans* |
|  | *Nocardioides flavus* |  | *Mycolicibacterium iranicum* |
|  | *Noviherbaspirillum canariense* |  | *Mycolicibacterium litorale* |
|  | *Rhodococcus cercidiphylli* |  | *Rhodococcus cercidiphylli* |
|  | *Skermanella rosea* |  | *Rhodococcus pedocola* |
|  | *Streptomyces exfoliatus* |  | *Skermanella aerolata* |
|  |  |  | *Sphingomonas zeicaulis* |
|  |  |  | *Streptomyces chryseus* |
|  |  |  | *Xanthobacter flavus* |
| 5 | *Kocuria flava* |  | *Massilia agri* |
|  | *Mycobacterium anyangense* |  | *Methylobacterium phyllostachyos* |
|  | *Mycobacterium arabiense* |  | *Methylorubrum populi* |
|  | *Noviherbaspirillum soli* |  | *Mycobacterium arabiense* |
|  | *Pseudomonas songnenensis* |  | *Noviherbaspirillum denitrificans* |
|  | *Sphingobium naphthae* |  | *Skermanella aerolata* |
|  | *Sphingomonas zeicaulis* |  | *Streptomyces kalpinensis* |
| 6 | *Gordonia didemni* |  | *Methylorubrum podarium* |
|  | *Kocuria polaris* |  | *Microbacterium aureliae* |
|  | *Methylobacterium phyllostachyos* |  | *Mycobacterium arabiense* |
|  | *Mycolicibacterium iranicum* |  | *Mycobacterium farcinogenes* |
|  | *Nocardia fluminea* |  | *Mycobacterium hackensackense* |
|  | *Nocardioides flavus* |  | *Mycolicibacterium litorale* |
|  | *Streptomyces abikoensis* |  | *Nocardia cyriacigeorgica* |
|  | *Streptomyces fumanus* |  | *Nocardia fluminea* |
|  | *Streptomyces kalpinensis* |  | *Nocardia takedensis* |
|  | *Williamsia marianensis* |  | *Rhodococcus pedocola* |
|  |  |  | *Sinorhizobium meliloti* |
|  |  |  | *Streptomyces alanosinicus* |
|  |  |  | *Streptomyces purpurascens* |
|  |  |  | *Williamsia marianensis* |

**Table S4.** Minor hydrocarbonoclastic bacteria in the desert soil samples polluted with different concentrations of crude oil at time zero and after 6 months (shaded areas in Pie charts in Figure 4).

| Crude oil concentration (%) | Incubation period (months) | |
| --- | --- | --- |
|  | 0 | 6 |
| 0 | *Enterobacter sichuanensis* | *Nocardioides ganghwensis* |
|  | *Micromonospora terminaliae* | *Nocardioides luteus* |
|  |  | *Nocardioides taihuensis* |
| 1 | *Bacillus muralis* | *Gordonia didemni* |
|  | *Kocuria turfanensis* | *Pseudarthrobacter polychromogenes* |
|  | *Micromonospora terminaliae* | *Streptomyces indoligenes* |
|  | *Pseudoxanthomonas japonensis* | *Streptomyces thermospinosisporus* |
|  | *Sinorhizobium meliloti* |  |
|  | *Streptomyces tibetensis* |  |
| 10 | *Arthrobacter ginsengisoli* | *Actinotalea ferrariae* |
|  | *Enterobacter sichuanensis* | *Agromyces indicus* |
|  | *Kribbella catacumbae* | *Arthrobacter ginsengisoli* |
|  | *Paenarthrobacter nitroguajacolicus* |  |
|  | *Paenibacillus xylanexedens* |  |
|  | *Pantoea dispersa* |  |
|  | *Streptomyces deserti* |  |
| 20 | *Acinetobacter johnsonii* | *Intrasporangium calvum* |
|  | *Kribbella catacumbae* | *Mesorhizobium thiogangeticum* |
|  | *Nocardioides luteus* |  |
|  | *Paenarthrobacter nicotinovorans* |  |
|  | *Streptomyces blastmyceticus* |  |
|  | *Streptomyces griseoruber* |  |
|  | *Streptomyces paludis* |  |
| 30 | *Arthrobacter gyeryongensis* |  |
|  | *Bacillus fumarioli* |  |
|  | *Bacillus halosaccharovorans* |  |
|  | *Kribbella sandramycini* |  |
|  | *Streptomyces chryseus* |  |
|  | *Streptomyces griseoflavus* |  |
|  | *Streptomyces indoligenes* |  |
